# Familial load of psychiatric disorders and overall functioning in patients newly diagnosed with bipolar disorder and their unaffected first-degree relatives

**DOI:** 10.1186/s40345-022-00277-1

**Published:** 2022-12-05

**Authors:** Kimie Stefanie Ormstrup Sletved, Katrine Maiggaard, Anne Amalie Elgaard Thorup, Lars Vedel Kessing, Maj Vinberg

**Affiliations:** 1 Copenhagen Affective Disorder Research Center (CADIC), Psychiatric Center Copenhagen, Copenhagen, Denmark; 2 Department of Clinical Medicine, University of Copenhagen, Copenhagen, Denmark; 3 Child and Adolescent Mental Health Center, Capital Region of Denmark, Copenhagen, Denmark; 4 Northern Zealand, Mental Health Center, Copenhagen University Hospital, Mental Health Services CPH, Copenhagen, Denmark

## Abstract

**Background:**

Overall functioning is already impaired in patients newly diagnosed with bipolar disorder (BD) and, to a lesser degree, also in their unaffected first-degree relatives (UR). Further, aggregation of psychiatric disorders among the patients’ first-degree relatives seems to be associated with higher illness burden and poorer prognosis. However, whether this aggregation of psychiatric disorders among first-degree relatives, the familial load (FL), impacts overall functioning in patients newly diagnosed with BD and their UR remains unresolved.

**Methods:**

In total, 388 patients newly diagnosed with BD, 144 of their UR and 201 healthy control individuals were included. Overall functioning was assessed using three different assessment methods: The interviewer based “Functioning Assessment Short Test” (FAST), the questionnaire “Work and Social Adjustment Scale” (WSAS) and six outcome measures covering the participants’ socio-economic status (SES); educational achievement, employment, work ability, relationship, cohabitation and marital status. Familial load of psychiatric disorder was assessed using the “Family History Research Diagnostic Criteria” interview. Associations between FL and overall functioning in patients and UR were investigated categorically using logistic and continuously in linear regression models.

**Results:**

Contrasting with the hypotheses, the FL of psychiatric disorders was not associated with impaired overall functioning, neither in patients newly diagnosed with BD nor in their UR.

**Conclusion:**

The findings indicate that impaired functioning in the early phase of BD is not associated with aggregation of psychiatric disorders among first-degree relatives. The observed functional impairment in patients newly diagnosed with BD seems driven by the personal impact of the disorder rather than the impact of having first-degree relatives with psychiatric disorders. Keywords: bipolar disorder, first-degree relatives, familial load of psychiatric disorders, functioning, socio-economic status.

## Introduction

Bipolar disorder (BD) is a major cause of disability worldwide, ranking with the fourth-highest disability adjusted life years (DALY) of all mental disorders [1]. Bipolar disorder affects overall functioning across the lifespan, and as the disorder often starts in youth and early adulthood, the overall illness burden has a long-term impact on the patients’ overall functioning, socio-economic status (SES) and their close relations [2–4].

The aetiology of BD is multifactorial, and evidence from twin, family, and adoption studies reveals a strong genetic predisposition [5, 6]. However, the associations between genotype and phenotype are complex and involve the interplay of genetic mechanisms and environmental factors [7]. Studies of familial high-risk individuals, like unaffected first-degree relatives, can provide insights into inherited vulnerability, influence of potential risk factors, determination of intermediate causal pathways, and identification of prodromal stages [8]. High-risk studies, therefore, offer pivotal opportunities to study risk factors before they are influenced by the effect of repeated mood episodes, medical treatment, and comorbidity. However, specific transmitted factors are not well discerned or defined; thus, a more integrative approach is needed [9].

In a nation-wide register-based study, patients with BD and, to a lower degree, also their unaffected full siblings presented with lower socio-economic status measured on educational achievement, employment status, personal income, cohabitation and marital status compared with control individuals from the general population [4]. Regarding the unaffected siblings, this is in line with results from a systematic review, reporting poorer functioning in first-degree relatives of patients with severe mental illness compared with control individuals with no first-degree relative with severe mental illness [10]. Furthermore, a recent study from our group revealed that overall functioning and socio-economic status (SES) were impaired already in the early course of BD, in patients diagnosed within the preceding two years and partly also in their unaffected siblings in comparison with healthy control individuals (HC) [11].

Due to high heritability, BD aggregates in families, and the aggregation of affected first-degree relatives can be investigated as the familial load (FL) of psychiatric disorders [12, 13]. Some individuals have no relatives with psychiatric disorders, reflecting a low FL (= 0), whereas other individuals can have several (two/three/four or even more) first-degree relatives with psychiatric disorders reflecting a high FL. Within BD, some studies have shown that higher FL was associated with poorer functioning [14, 15].

Nevertheless, whether FL of psychiatric disorders is associated with functioning in patients newly diagnosed with BD has not been investigated. Since this group of patients are at an early stage of their illness phase, FL may be differently associated with functioning. Moreover, since having a first-degree relative with severe mental illness seem to inflict functioning to some extent in unaffected individuals, it is of interest to elucidate if aggregation of psychiatric disorders (in two or three or more) among first-degree relatives is associated with increased functional impairment.

## Aims of the study

We aimed to investigate whether FL of psychiatric disorders among first-degree relatives was associated with the overall functioning patients newly diagnosed with BD and their UR.

### Hypotheses

We hypothesized that (i) higher FL of psychiatric disorders would be associated with a higher degree of impaired functioning in patients newly diagnosed with BD, (ii) higher FL of psychiatric disorders would be associated with a higher degree of impaired functioning in unaffected first-degree relatives of patients newly diagnosed with BD.

## Methods

### Study design

The present study is based on cross-sectional baseline data from the large-scale ongoing longitudinal clinical study, the Bipolar Illness Onset study (the BIO-study), initiated in 2015 [16].

### Included participants

#### Patients newly diagnosed with bipolar disorder

We invited patients aged 18–70 at the Copenhagen Affective Disorder Clinic at Psychiatric Centre Copenhagen. The Centre covers all psychiatric centres in the Capital Region of Denmark and provides assessments of and treatment for patients newly diagnosed with BD in the catchment area. Patients are assessed and diagnosed by specialists in psychiatry according to the ICD-10 and DSM criteria upon referral to the Copenhagen Affective Disorder Clinic. Upon referral to the Copenhagen Affective Disorder Clinic, patients were routinely invited to participate in the BIO-study if a diagnosis of BD was set within the preceding two years, as this was chosen as the definition of being “newly diagnosed”. Patients aged 15–18 were recruited from the highly specialized Bipolar Team of the Child and Adolescent Mental Health Center, Capital Region of Denmark.

#### Unaffected first-degree relatives

If included patients consented, we invited their eligible siblings and children to participate in the study. The inclusion criteria were age 15–70 years and having a full sibling or a parent newly diagnosed with BD participating in the BIO-study. Exclusion criteria were being diagnosed with BD or schizophrenia.

#### Healthy control individuals

Healthy control individuals were included from the blood bank at Rigshospitalet in Copenhagen, Denmark. Random blood donors were approached on random days upon blood donation and invited to participate in the study. Inclusion criterion was age 15–70 years. Exclusion criteria were a current or previous medically treated psychiatric disorder in the subject or a first-degree relative.

#### Diagnostic and clinical assessments

Participants were included from June 2015 to January 2021. Following informed consent for participation, a PhD student (medical doctor or a psychologist) confirmed the BD diagnosis using the Schedules for Clinical Assessment in Neuropsychiatry (SCAN) at inclusion in the study [17]. Similarly, UR and HC underwent a SCAN interview. Clinical assessments for depressive and manic symptoms were performed using the Hamilton Depression Scale-17 (HDRS-17) and the Young Mania Rating Scale (YMRS) [18, 19].

#### Assessment of familial load of psychiatric disorders

Using a translated and modified Danish version of Family History Research Diagnostic Criteria (FH-RDC) [20], each participant was systematically interviewed about the presence of any current or previous psychiatric disorder in their first-degree relatives to the best of their knowledge. Information on parents, siblings and offspring were registered in six categories (0 = no psychiatric disorder, 1 = depression and/or suicide, 2 = BD, 3 = affective disorder not specified, 4 = schizophrenia, 5 = alcohol abuse, 6 = other psychiatric disorder (e.g., anxiety, autism or ADHD). Additionally, information on consummated suicides in first-degree relatives was registered. If a participant was uncertain about family members’ psychiatric symptoms or disorders, we conservatively noted the status of that family member as “0”, no psychiatric disorder. In cases where the patient was not genetically related to their first-degree relatives, e.g., due to adoption, the FH-RDC interview was not performed.

#### Estimating familial load (FL)

Different approaches have previously been used to analyze FL. Some studies have listed the number of family members with psychiatric disorders categorically, either as an ordinal categorical variable (number of diseases family members: 0, 1, 2, 3, 4+) [15] or a binary categorical variable (diseased family members: “yes”/“no”) [12, 14]. In contrast, other studies have argued that analyzing FL categorically might not capture the effect of FL on psychiatric disorders [21, 22]. It has therefore been suggested that a continuous “familial loading score” should be estimated and used for analyses of FL [23]. To ensure that we did not overlook a true association, we investigated FL in two ways: as a binary categorical and as a continuous variable.

#### Familial load as a categorical variable

Familial load was analyzed as a binary categorical variable. In patients, we investigated if having ≥ 1 first-degree relative with a psychiatric disorder was associated with impaired functioning compared with patients with no first-degree relative with a psychiatric disorder.

All included UR had at least one first-degree relative with BD, this being the proband patient. Therefore, in the UR group, we investigated if having ≥ 2 first-degree relatives with psychiatric disorders was associated with impaired functioning compared with UR, with only 1 first-degree relative with a psychiatric disorder.

#### Familial load as a continuous variable

Additionally, FL was analyzed as a continuous variable, measuring the total load of psychiatric disorders among first-degree relatives. We used the Family Liability Index (FLI), which has also been used to analyze FL in high-risk individuals [24]:

$${\rm{Family\, Liability\, Index }}({\rm{FLI}}){\rm{ }} = \frac{{{\rm{BM}} + {\rm{BF}} + \sum {\rm{BS }} + {\rm{ }}(\sum {\rm{HS}} \cdot 0.5)}}{{2 + n\;{\rm{BS}} + (n\;{\rm{HS}} \cdot 0.5)}}{\rm{ }}.$$

The Family Liability Index (FLI) reflects the aggregation of psychiatric disorders among a participant’s first-degree relatives. The FLI is estimated based on the presence of psychiatric disorders in the biological mother (BM), the biological father (BF), biological siblings (BS) and biological half-siblings (HS). The index considers that half-siblings share only half the genetic information compared to parents and full siblings. The estimate indicates the load of diseased first-degree relatives in the numerator *compared* to the number of first-degree relatives (the family size) in the denominator. The FLI continuously assumes values from 0 to 1, with 0 reflecting “low familial load” and 1 reflecting “high familial load”.

#### Assessment of functioning

Functioning may be measured and estimated in various ways. In this study, we investigated functioning in three ways, as presented in the following.

#### The Functioning Assessment Short Test (FAST)

The FAST is a clinical observer-based interview to assess the participants’ overall functioning in the previous two weeks. It covers the subdomains of autonomy, occupation, cognition, financial issues, interpersonal relationships and leisure time. Total scores range from 0 to 72, the higher score, the more significant functional impairment [25]. FAST total scores between 0 and 11 indicate no impairment, 12–20 indicate mild impairment, 21–40 indicate moderate impairment and FAST total scores above 40 indicate severely impaired functioning [26].

#### The work and Social Adjustment Scale (WSAS)

The WSAS is a self-reported questionnaire in five subitems. The questionnaire covers work ability, practical housework, participating in social activities, having meaningful leisure time, and engaging in social relations. Participants rate their everyday life functioning from 0 to 8 on each item. Total scores range from 0 to 40, the higher score, the more significant impairment in self-reported functioning. The WSAS is sensitive in patients with BD and individuals without psychiatric disorders [27–29].

#### Socio-economic status (SES)

At baseline assessment, all participants reported on the following six domains of SES:

1. Educational achievement; measured continuously in total years of education.
2. Employment status; measured as “employed, student, pension or other” vs. “unemployed or disabled”.
3. Work ability; measured as “not on sick-leave” vs. “on sick-leave”.
4. Relationship status; measured as “being in a relationship” vs. “not in a relationship”.
5. Cohabitation status; measured as “living with someone in terms of shared address” vs. “living alone”.
6. Marital status; measured as “married, divorced, separated or widowed” vs. “never married”.

These six socio-economic domains were adopted from Statistics Denmark as proxies of overall SES [30] and have been used previously [4].

### Statistical analyses

Descriptive data were analyzed to test assumptions of normal distribution. Continuous outcomes were analyzed in the student’s t-test, and categorical outcomes were analyzed using chi-square tests for pairwise comparisons of BD vs. HC and UR vs. HC, respectively. The correlations between FLI and FAST, WSAS and educational length were explored using 2-tailed Spearman’s correlation tests. The associations between FL and functioning were analyzed with multiple regression models with the continuous outcomes (FAST (total score and subdomains), WSAS and educational achievement and with binary logistic regression models with categorical outcomes (employment status, work ability, relationship, cohabitation and marital status). We performed analyses unadjusted (model 1) and adjusted for age, sex, HDRS-17 and YMRS (model 2). All analyses were performed separately on all outcomes on patients with BD and on UR. As multiple analyses were conducted, a Bonferroni correction for multiple testing was applied and the adjusted significance level was p < 0.00625. The Statistical Package for Social Sciences (SPSS Statistics 25) was used, and all model assumptions were met.

## Results

We included 388 patients newly diagnosed with BD, 144 of their unaffected first-degree relatives and 201 healthy control individuals. Of the 144 UR, 131 (91%) were siblings of proband BD patients, and the remaining 13 (9%) were included as offspring of parents with BD. Demographic and functional outcomes are presented in Table 1. In the pairwise comparisons, patients with BD had statistically significant impaired functioning compared with the HC in FAST (mean difference: 20.5 [95%CI: 19.0–21.9] p < 0.001), WSAS (mean difference: 17.5 [95% CI: 16.4–18.6] p < 0.001), and in five out of six SES measures. The UR presented with impaired functioning in FAST (mean difference: 4.8 [95% CI: 3.1–6.4] p < 0.001), WSAS (mean difference: 3.1 [95% CI: 1.6–4.5] p < 0.001), and in one of the six SES measures; the educational achievement (mean difference: − 1.2 years [95%CI: − 0.7 to (− 1.7)] p < 0.001) compared with the HC.

**Table 1:** Clinical, functional and socio-economic outcomes in patients newly diagnosed with bipolar disorder (BD), their unaffected first-degree relatives (UR) and healthy control individuals (HC)

	BD	UR	HC
Number (N)	388	144	201
Sex (% female)	249 (64.2%)	77 (53.5%)*	131 (65.2%)
Age, median [IQR]	28.4 [23.6; 36.5]	27.0 [22.4; 35.3]	27.3 [24.0; 35.9]
HDRS-17, median [IQR]	9 [5; 15]*	2 [0; 3]*	0 [0; 2]
YMRS, median [IQR]	3 [0; 7]*	0 [0; 2]*	0 [0; 1]
Functioning Assessment Short Test (FAST) mean (SD), N	380	143	200
FAST total score	22.0 (13.7)*	6.3 (9.7)*	1.5 (2.6)
FAST subdomains			
Autonomy	2.8 (2.9)*	0.8 (1.8)*	0.2 (0.6)
Occupation	6.9 (6.3)*	1.5 (3.6)*	0.2 (1.2)
Cognition	5.0 (3.7)*	1.4 (2.1)*	0.5 (0.9)
Finances	1.5 (1.9)*	0.3 (0.8)*	0.1 (0.4)
Relations	4.0 (3.6)*	1.4 (2.4)*	0.4 (1.0)
Leisure time	1.8 (1.8)*	0.8 (1.3)*	0.2 (0.5)
Work and Social Adjustment Scale (WSAS) mean (SD), N	380	143	200
WSAS, total score	19.6 (7.8)*	5.3 (7.4)*	2.2 (4.8)
Socio-economic status, N (%)	381	140	196
Educational years, mean (SD)	14.4 (2.7)*	14.3 (2.6)*	15.6 (2.0)
Employment	290 (74.9%)*	128 (88.9%)	173 (86.1%)
Work ability	297 (76.7%)*	142 (98.6%)	200 (99.5%)
Relationship	188 (48.6%)*	85 (59.0%)	132 (65.7%)
Cohabitation	249 (64.3%)*	102 (70.8%)	146 (72.6%)
Marital status	96 (24.8%)	37 (25.7%)	38 (18.9%)

Continuous variables are presented as median [Interquartile range] or mean (standard deviation)

Categorical variables are presented as N (%)

HDRS-17: The Hamilton Depression Rating Scale, total score

YMRS: The Young Mania Rating Scale, total score

Education: highest obtained educational achievement, measured in total years

Employment: participants in job/studying

Work ability: participants not on sick-leave

Relationship: participants with a partner

Cohabitation: participants living with someone

Marital status: participants married, separated, divorced or widowed

*Statistical significant differences in pairwise comparisons of BD compared with HC and UR compared with HC

Table 2 presents the FL of psychiatric disorders in patients newly diagnosed with BD and their UR. Half of the patients newly diagnosed with BD (50.1%) had a parent with a psychiatric disorder, and 64.9% had one or more first-degree relatives with psychiatric disorders. This comprised 33.5% with a first-degree relative with depression and/or suicide, 15.5% with BD, 0.3% with affective disorder not specified, 1.5% with schizophrenia, 12.4% with alcohol abuse and 16.8% with other known psychiatric disorders, such as anxiety, autism and ADHD. Due to the inclusion criterion, all included UR (100%) had at least one first-degree relative with a psychiatric disorder, this being the proband patient with BD. Additionally, 45.1% had two or more first-degree relatives with psychiatric disorders, comprising 21.5% with depression, 0.7% with schizophrenia, 11.1% with alcohol abuse and 8.3% with other known psychiatric disorders.

**Table 2:** Familial load (FL) of psychiatric disorders (PD) in patients newly diagnosed with bipolar disorder (BD) and their unaffected first-degree relatives (UR)

	BD	UR
Number, N	387	143
≥ 1 parent with PD	194 (50.1%)	62 (43.1%)
≥ 1 sibling with PD	104 (26.9%)	135 (93.8%)
≥ 1 offspring with PD	13 (3.4%)	4 (2.8%)
≥ 1 first-degree relative with PD	251 (64.9%)	143 (100%)
≥ 2 first-degree relatives with PD	90 (23.3%)	64 (45.1%)
Number of first-degree relatives with PD		
0	136 (35.1%)	0
1	161 (41.6%)	79 (55.2%)
2	66 (17.1%)	53 (37.1%)
3	22 (5.7%)	10 (7.0%)
4	0	1 (0.7%)
5	1 (0.3%)	0
6	1 (0.3%)	0
First-degree relatives with PD		
Depression and/or suicide	130 (33.5%)	31 (21.5%)
Bipolar disorder	60 (15.5%)	144 (100%)^a^
Affective disorder, not other specified	1 (0.3%)	0
Schizophrenia	6 (1.5%)	1 (0.7%)
Alcohol abuse	48 (12.4%)	16 (11.1%)
Other known psychiatric disorder	65 (16.8%)	12 (8.3%)
Suicide		
Consummated suicide in a first-degree relative	9 (2.3%)	6 (4.2%)
Family Liability Index (FLI)		
FLI	0.32 (0.30)	0.45 (0.22)

Categorical variables are presented as N (%)

Continuous variables are presented as mean and standard deviation (SD)

≥ 1 PD Relative: Participants with one or more first-degree relative with a psychiatric disorder

≥ 2 PD Relatives: Participants with two or more first-degree relatives with psychiatric disorders

^a^ All UR have one or more relatives with bipolar disorder due to the inclusion criteria

### Correlations between family liability index and functioning

**Fig. 1:**
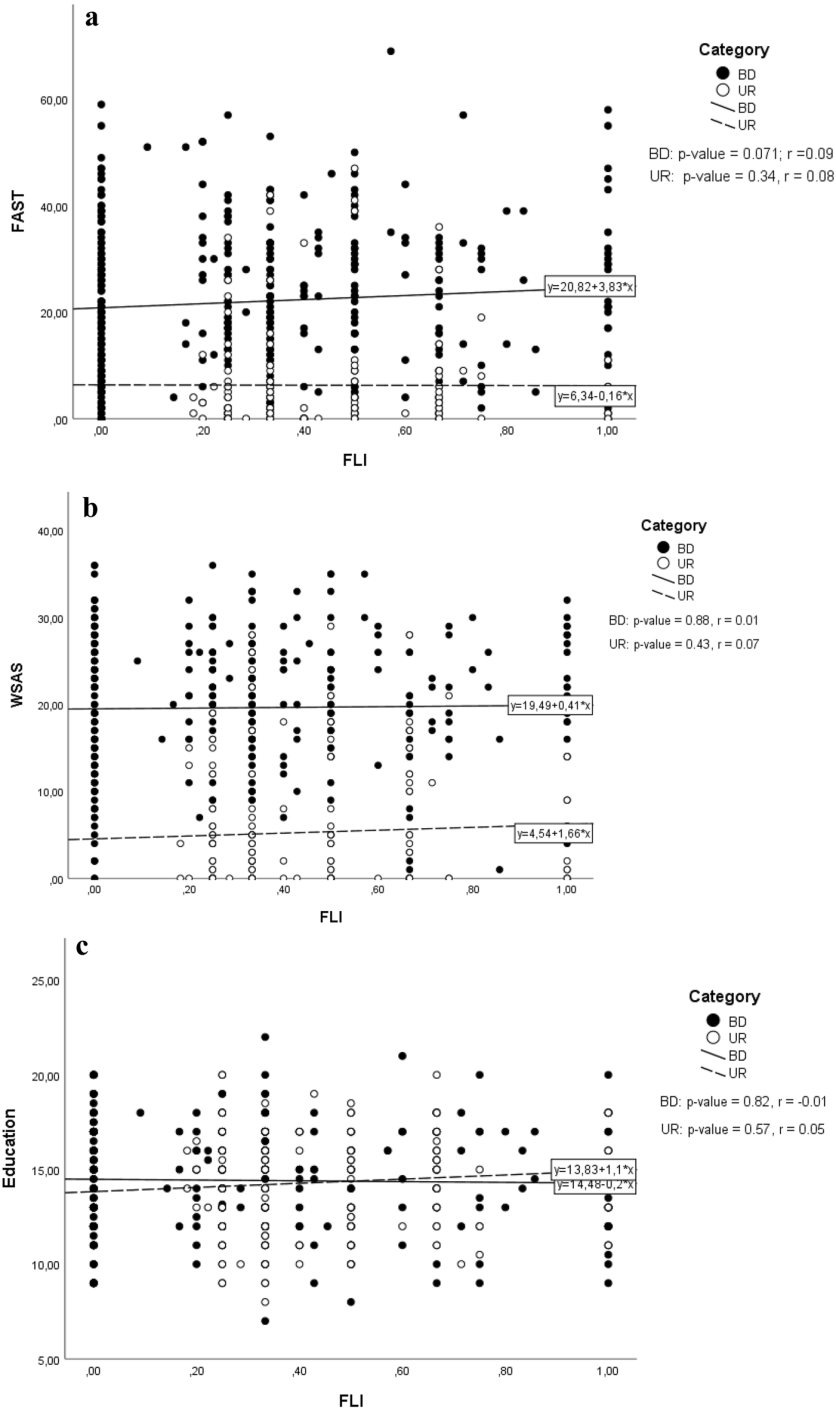
Associations between Family Liability Index (FLI) and** a** Functioning Assessment Short Test (FAST),** b** Work and Social Adjustment Scale (WSAS) and** c** Educational achievement (years of education) in 388 patients newly diagnosed with bipolar disorder (BD) and 144 of their unaffected first-degree relatives (UR)

Figure 1a, b, and c present correlations between the continuous variable Family Liability Index (FLI) and FAST, WSAS and educational achievement in patients with BD and their UR. The strongest correlation was found between FLI and FAST in patients (r: 0.09 and p = 0.071) in Spearman’s correlation test, yet none of the tests revealed a correlation between FLI and functioning reaching statistical significance.

### Associations between familial load and functioning

In Table 3, in multiple regression analyses, we found no statistically significant associations between FL and FAST (total scores or subdomain-scores), WSAS, or educational achievement in patients with BD or their UR.

**Table 3:** Associations between familial load of psychiatric disorders (PD) and the outcomes (1) Functioning Assessment Short Test (FAST) in total scores and subdomains, (2) Work and Social Adjustment scale (WSAS) and (3) Years of education in patients newly diagnosed with bipolar disorder (BD) and their unaffected first-degree relatives (UR).

Patients newly diagnosed with BD (n = 388)				UR of patients newly diagnosed with BD (n = 144)			
	B	95% CI	P		B	95% CI	P
FAST, total score							
≥ 1 PD Relative	1.66	[− 0.67; 3.98]	0.162	≥ 2 PD Relatives	0.33	[− 2.62; 3.27]	0.828
FLI	2.31	[− 1.42; 6.04]	0.223	FLI	− 3.12	[− 9.69; 3.26]	0.328
FAST subdomains							
Autonomy							
≥ 1 PD Relative	0.32	[− 0.20; 0.85]	0.229	≥ 2 PD Relatives	0.16	[− 0.40; 0.72]	0.572
FLI	0.42	[− 0.42; 1.27]	0.325	FLI	− 0.30	[− 1.5; 0.94]	0.634
Occupation							
≥ 1 PD Relative	0.55	[− 0.70; 1.81]	0.387	≥ 2 PD Relatives	− 0.55	[− 1.69; 0.60]	0.347
FLI	0.50	[− 1.51; 2.52]	0.623	FLI	− 2.07	[− 4.57; 0.44]	0.105
Cognition							
≥ 1 PD Relative	0.21	[− 0.45; 0.87]	0.532	≥ 2 PD Relatives	− 0.16	[− 0.82; 0.51]	0.641
FLI	0.41	[− 0.65; 1.46]	0.449	FLI	− 0.99	[− 2.45; 0.46]	0.180
Finances							
≥ 1 PD Relative	0.20	[− 0.18; 0.57]	0.299	≥ 2 PD Relatives	0.03	[− 0.24; 0.30]	0.824
FLI	0.08	[− 0.52; 0.67]	0.804	FLI	− 0.22	[− 0.80; 0.37]	0.461
Relations							
≥ 1 PD Relative	0.28	[− 0.36; 0.91]	0.392	≥ 2 PD Relatives	0.58	[− 0.19; 1.36]	0.140
FLI	0.82	[− 0.20; 1.84]	0.113	FLI	0.01	[− 1.72; 1.73]	0.991
Leisure							
≥ 1 PD Relative	0.10	[− 0.23; 0.43]	0.560	≥ 2 PD Relatives	0.26	[− 0.16; 0.67]	0.230
FLI	0.08	[− 0.45; 0.62]	0.759	FLI	0.35	[− 0.57; 1.23]	0.453
WSAS							
≥ 1 PD Relative	− 0.04	[− 1.56; 1.50]	0.956	≥ 2 PD Relatives	1.14	[− 1.22; 3.50]	0.342
FLI	− 0.51	[− 2.96; 1.94]	0.683	FLI	0.74	[− 4.53; 6.00]	0.782
Education							
≥ 1 PD Relative	− 0.15	[− 0.67; 0.38]	0.589	≥ 2 PD Relatives	− 0.17	[− 1.05; 0.72]	0.709
FLI	− 0.20	[− 1.05; 6.64]	0.639	FLI	0.89	[− 1.04; 2.82]	0.363

Analyses presented are adjusted to age, sex, HDRS-17 and YMRS

HDRS-17: Hamilton Depression Rating Scale, total score

YMRS: Young Mania Rating Scale, total score

FAST: Functioning Assessment Short Test

WSAS: Work and Social Adjustment Scale

Education: educational achievement measured in years

≥ 1 PD Relative: Participants with one or more first-degree relatives with psychiatric disorders

≥ 2 PD Relatives: Participants with two or more first-degree relatives with psychiatric disorders

*FLI:* Family Liability Index

In Table 4, in logistic regression models, we found no statistically significant associations between FL and any of the five categorical SES measures in patients with BD or their UR. In the UR group, in the fully adjusted model for age, sex, HDRS-17 and YMRS, the FL was positively associated with higher odds of cohabitating on the continuous variable FLI (OR: 3.11 [95% CI: 1.25; 7.71] p = 0.014) and the categorical variable ≥ 2 relatives with a psychiatric disorder (OR: 10.98 [95% CI: 1.25; 96.70] p = 0.031). However, this finding of a protecting effect did not reach statistical significance at a bonferroni corrected significance level of p < 0.00625.

**Table 4:** Associations between familial load of psychiatric disorders (PD) and the outcomes (1) Employment, (2) Work ability, (3) Relationship, (4) Cohabitation and (5) Marital status in patients newly diagnosed with bipolar disorder (BD) and their unaffected first-degree relatives (UR).

Patients newly diagnosed with BD (n = 388)				UR of patients newly diagnosed with BD (n = 144)			
	OR	95% CI	P		OR	95% CI	P
Employment							
≥ 1 PD Relative	0.77	[0.47; 1.27]	0.299	≥ 2 PD Relatives	1.14	[0.32; 4.11]	0.839
FLI	1.82	[0.79; 4.20]	0.163	FLI	5.43	[0.22; 136.1]	0.303
Work ability							
≥ 1 PD Relative	1.18	[0.71; 1.96]	0.531	≥ 2 PD Relatives	–	–	–
FLI	0.92	[0.41; 2.08]	0.847	FLI	–	–	–
Relationship							
≥ 1 PD Relative	1.26	[0.81; 1.96]	0.313	≥ 2 PD Relatives	1.15	[0.55; 2.41]	0.710
FLI	1.06	[0.52; 2.16]	0.866	FLI	4.66	[0.80; 27.28]	0.088
Cohabitation							
≥ 1 PD Relative	1.11	[0.71; 1.73]	0.646	≥ 2 PD Relatives	3.11	[1.25; 7.71]	0.014
FLI	1.16	[0.57; 2.39]	0.683	FLI	10.98	[1.25; 96.70]	0.031
Marital status							
≥ 1 PD Relative	0.88	[0.46; 1.68]	0.693	≥ 2 PD Relatives	2.30	[0.77; 6.89]	0.136
FLI	1.08	[0.40; 2.91]	0.874	FLI	3.03	[0.32; 29.20]	0.337

Analyses presented are adjusted to age, sex, HDRS-17 and YMRS

HDRS-17: Hamilton Depression Rating Scale, total score

YMRS: Young Mania Rating Scale, total score

Employment: patients “in job/study” vs. “unemployed”

Work ability: patients “not on sick-leave” vs. “on sick-leave”

Relationship: patients “in a relationship” vs. “not in a relationship”

Cohabitation: patients “cohabitating” vs. “living alone”

Marital status: patients “married, divorced or separated” vs. “never married”

≥ 1 PD Relative: Participants with one or more first-degree relatives with psychiatric disorders

≥ 2 PD Relatives: Participants with two or more first-degree relatives with psychiatric disorders

*FLI:* Family Liability Index.

### Post hoc exploratory analyses

As primary analyses did not reveal any statistically significant associations in unadjusted or adjusted models, post hoc exploratory analyses were performed to explore possible associations on subgroup levels. First, we investigated whether restriction to severe mental illnesses (depression, suicide, BD or schizophrenia) instead of all psychiatric disorders in first-degree relatives was associated with functioning. We found no statistically significant associations between FL of severe mental illness and any outcome measures of functioning in patients or their UR. Second, within patients, we investigated associations between an FL, specifically with BD diagnosis in a first-degree relative and functioning. There were no statistically significant associations between a familial and any measure of functioning in the 15.5% of patients with a first-degree relative with BD compared with the 84.5% without a first-degree relative with BD.

As the impact of a familial predisposition to psychiatric disorders could be increased in the patients who were offspring of a parent with a psychiatric disorder, the associations between parental psychiatric disorder and functioning were explored. Within BD and UR, separate analyses were performed on participants with vs. without a parent with a psychiatric disorder. Patients with a parent with a psychiatric disorder presented with numerically higher FAST total scores compared with patients without a parent with a psychiatric disorder, however the difference did not reach statistical significance (mean difference: 2.59, [95% CI: − 0.17 to 5.34]; p = 0.066). No associations were seen in the UR group when analyzing the group of UR having a parent with psychiatric disorders separately.

Lastly, we performed sensitivity analyses on the UR group restricted to include the 131 UR who were siblings of a patient with BD, since FL potentially could be differently associated with functioning in siblings of patients with BD compared with in offspring of a parent with BD. No statistically significant associations between FL and functioning were seen in sensitivity analyses of siblings of patients with BD.

## Discussion

This study investigated the associations between aggregation of psychiatric disorders among first-degree relatives and overall functioning in 388 patients newly diagnosed with BD and 144 of their UR. In contrast with our hypotheses, neither the patients with BD nor their UR presented with a higher degree of functional impairment in the case of more first-degree relatives with psychiatric disorders.

### Comparison with prior studies on familial load in BD

In contrast to these findings, previous studies have described that a higher FL was associated with earlier onset, poorer clinical outcomes, and poorer treatment response in patients with BD [14, 15, 31]. However, most of these studies did not report on overall functioning as the outcome, and only one study investigated the association between the FL of severe mental illness and patients’ SES [15]. The latter study included 757 American outpatients with BD and found that high FL was associated with having more children, lower educational achievement, and a lower household income but not marital status [15]. The contrasting findings may be explained by the difference in age. The patients’ mean age was 39.1 years, and illness duration was 19.9–25.9 years in the American study [32, 33] versus a median age of 28.4 year and an illness duration of 10 [IQR: 5–16] years in the present study [11]. In this study, the patients’ overall functioning was less impaired, most likely because they were newly diagnosed with BD, earlier in their illness course and included at an early stage of the BD illness. Since the patients included in this study were younger, they may also have a lower FL of psychiatric disorders as their first-degree relatives have had fewer cumulative years at risk of developing psychiatric disorders [34]. Moreover, the other study was conducted on American outpatients, limiting the direct comparability of SES since there may be vast differences between American and Danish (European) patients with BD [35]. Lastly, the 757 patients in the American study were pooled from two randomized controlled trials [32, 33], and patients were excluded if they, for example, did not tolerate or respond to lithium and quetiapine, which decreases the generalizability and comparability of the findings [15].

### Prior studies on familial load in unaffected first-degree relatives of patients with BD

Several studies within familial high-risk individuals have investigated FL as a risk factor for developing psychiatric illness [12, 13]. Furthermore, some studies have investigated functioning and SES in familial high-risk individuals compared with control individuals or low-risk individuals [36]. However, no previous study has systematically investigated whether higher FL was associated with overall functioning in UR of patients with BD.

In the before mentioned studies, the nation-wide register-based study [4] and the clinical cross-sectional study [11], we showed an overall impaired functioning in full siblings of patients with BD compared with control individuals. This indicates that overall functioning may partly be a familial related trait. Findings in this study may suggest that impaired functioning observed in the UR group may not be due to familial aggregation of psychiatric disorders but may be caused by other factors, e.g., minor psychiatric disorders or subthreshold psychiatric symptoms.

### Psychiatric disorders in specific family members

In the present study, parents and siblings were included as first-degree relatives, and a familial high-risk study has shown overall equal clinical vulnerability in siblings compared with offspring [37]. However, crucial environmental factors may differ between siblings and offspring of patients with mental illness [38], not least because parents tend to resemble each other phenotypically due to assortative or nonrandom mating [39, 40].

Therefore, the effects of FL may differ between being a full sibling to a proband with BD or an offspring of a parent with BD. As the median illness duration from the first affective episode was 10 years prior to the diagnosis, the close family members may have been exposed to the environmental stress of having an ill family member long before the diagnosis. Growing up with a parent with BD or a parent developing BD may impact the offspring’s functioning and mental health more broadly and to a higher degree than growing up with a sibling with BD since parents play a central role in family dynamics and as caregivers for their offspring during their upbringing. Our subgroup analyses indicated this pattern at a trend level, with a higher total FAST score in the UR with a parent with a psychiatric disorder compared with the UR with healthy parents.

## Strengths and limitations

The study participants consisted of three clinical well-described samples of medium sample size: the patients newly diagnosed with BD, their UR and age and sex-matched HC. All participants underwent thorough and systematical interviews, and functioning was thoroughly assessed using three different assessment tools. It is a strength that FL was estimated and analyzed in two ways; as a dichotomous (+/-) variable and as a continuous variable (FLI), to ensure we did not fail to observe a true association. In terms of including a representative patient population, we may have included a more heterogenous and complete sample to represent the total number of people living with BD when including patients who were recently diagnosed. In this way, the included patient population seems less selected and biased. First, the patient population represent patients at the time of diagnosis of BD. The population is younger than described in other studies, making it likely that the patients’ overall functioning is higher. Further, age in an important factor to consider when evaluating the effects of FL, since higher age is associated with higher FL [12]. Conclusively, when the patients are newly diagnosed and younger, their UR are also younger, making the actual FL more precisely estimated at the time of the patient’s diagnosis.

Functioning was assessed in three different ways: as socio-economic outcome domains, using a clinical rating-scale and a self-reported questionnaire in three groups with broad age distributions. Since functioning and socio-economic status may differ according to age, adjustments for age on all outcomes is important. To investigate associations of trends, it serves as a strength to have included participants in such wide age-spans (15–70 years). We made no attempt to test for associations with age-at-onset or with age in general as individuals divided into age spans, as most of the included patients were quite young (median age of 28.4 years [IQR: 23.6; 36.5]). Included patients were between 15 and 64 years old and only 20 (5.2%) of the included patients were above the age of 50 years.

Furthermore, some limitations do apply. Although each patient newly diagnosed with BD was systematically interviewed about the presence of any current or previous psychiatric disorder in their first-degree relatives using the Family History Research Diagnostic Criteria (FH-RDC) [20], we did not include interviews with first-degree relatives themselves except for the included UR. Hence, the present study did not include clinically evaluated diagnoses among all first-degree relatives. This way of assessing FL could potentially lead to an underestimation since mental health problems may be concealed or at least not discussed in some families. Second, the sample sizes may be a limitation. In particular, the UR group could be vulnerable to a type II error with 144 participants. However, the weak associations found between FL and SES in the UR group pointed toward a *protecting* effect of a higher FL leading to higher rates of cohabitation, being in a relationship and being married. Considering the direction of this weak finding, that contrasted with the hypotheses, we find it unlikely that a larger sample size would have shown the opposite and could have verified our hypotheses. Finally, not all eligible first-degree relatives were included in the study. Some because their proband relative with BD did not consent to invite them for participation, others because they did not accept the invitation to participate in the study. Conclusively, a risk of selection bias is indisputable.

## Conclusion

In patients newly diagnosed with BD and their unaffected first-degree relatives, familial load of psychiatric disorders among the patients and their UR was not associated with an overall functional impairment or lower socio-economic status. The present findings suggest that overall functioning in the early course of bipolar disorder may be adaptable and independent of an aggregation of psychiatric disorders among first-degree relatives. The impaired functioning seen in the UR group seems driven by factors other than having first-degree relatives with a psychiatric disorder, for example, partly driven by higher frequencies of psychiatric disorders in these high-risk individuals.

The loss of functioning in the early stages of BD emphasizes that early detection, monitoring, and treatment should be integrated into future treatment plans to preserve, strengthen, and prevent loss of functioning.

## Data Availability

Research data is not shared.
